# Systematization of peer review in *Epidemiologia e Serviços de Saúde*

**DOI:** 10.1590/S2237-96222024v33e20241001.en

**Published:** 2024-12-13

**Authors:** Marcus Tolentino Silva, Taís Freire Galvão

**Affiliations:** 1 Universidade de Brasília, Faculdade de Ciências da Saúde, Departamento de Saúde Coletiva, Brasília, DF, Brasil; 2 Universidade Estadual de Campinas, Faculdade de Ciências Farmacêuticas, Campinas, SP, Brasil; 3 Ministério da Saúde, Secretaria de Vigilância em Saúde e Ambiente, Coordenação-Geral de Editoração Técnico-Científica em Vigilância em Saúde, Brasília, DF, Brasil

Peer review is one of the pillars of quality and credibility in scientific. Despite its relevance, the process is imperfect and affected by variability in quality and delays. The process of peer review requires constant management of biases and inconsistencies. Certain factors directly influence publication quality, such as editorial screening and the number of reviewers ^1^. Additional reviews after acceptance can enhance scientific rigor but may also frustrate authors’ expectations. Detailed reviews are valued for the perception of quality they provide ^2^, though excessive steps delay publication ^3^.

Ineffective editorial processes lead to unjustifiable delays, such as the weeks-long wait for initial communications from some journals ^4^. Communication is often hindered by diffuse responsibilities among editors, resulting in misunderstandings in interactions with authors ^5^. Reducing timelines, improving communication, and providing constructive feedback increase author satisfaction ^6^. Enhancing the peer review process aims to reduce these information asymmetries, mitigate reviewer workload, and recognizes the contributions of those who effectively support the editorial process.

To improve the peer review process at *Epidemiologia e Serviços de Saúde: revista do SUS* (RESS), a checklist with hierarchical items was developed to guide the construction of reviews and organize the evaluation of manuscripts submitted to RESS ^7^. The use of tools like this in peer review has the potential to increase transparency and systematize the process ^8^ ^,^ ^9^.

To develop the checklist, we started with the main reasons for rejection of scientific articles submitted to journals ^10^ and conducted a literature review to identify prior experiences that could provide the basic framework ^11^ ^-^ ^18^. The authors’ participation in events such as the International Congress of Peer Review and Scientific Publication (https://peerreviewcongress.org) also provided insights that helped evolve the tool.

The checklist was structured into critical, important, and desirable items ^7^ to assist reviewers in evaluating whether the research meets essential requirements, such as the relevance of the research question for the Unified Health System (*Sistema Único de Saúde*, SUS), the adequacy of the study design for the proposed investigation, the alignment of methods - epidemiological, statistical, or qualitative - with the objective, the consistency and robustness of the results with the methods used, and the compatibility of the conclusion with the guiding question and SUS guidelines. The expectation is that its systematic adoption will benefit reviewers, editors, authors, and readers by organizing the review process to reduce bias and shorten evaluation time.

In order to test the checklist and train reviewers and editors, we organized the first ‘RESSathon: RESS peer review marathon’ in June 2024 in Brasília, conducted as an outreach project by the University of Brasília ^19^ ^).^ Similar review marathons have been reported to empower graduate students by engaging them in review processes and enhancing their skills in article evaluation ^20^. Two additional marathons were held in 2024, training a total of 96 researchers in peer review practices ^21^ ^,^ ^22^. These marathons can be replicated in other institutions interested in strengthening scientific communication among researchers, faculty, and graduate students, as well as bringing the academic community closer to RESS and its team. In addition to contributing to journals, serving as a reviewer promotes the development of critical skills and supports the consolidation of scientists’ careers ^23^. These efforts aim to strengthen the RESS editorial process, making it increasingly fair, effective, and aligned with SUS interest.
