# Supplementary material for: Systematization of peer review in Epidemiologia e Serviços de Saúde
Source: Epidemiol Serv Saude. 2024 Dec 13;33:e20241001. doi: 10.1590/S2237-96222024v33e20241001.en (PMC11654714; doi:10.1590/S2237-96222024v33e20241001.en)
Supplement: Translation

# Translation: Sistematização da revisão por pares na Epidemiologia e Serviços de Saúde

**DOI:** 10.1590/S2237-96222024v33e20241001.pt

**Authors:** Marcus Tolentino Silva, Taís Freire Galvão

**Affiliations:** 1 Universidade de Brasília, Faculdade de Ciências da Saúde, Departamento de Saúde Coletiva, Brasília, DF, Brasil; 2 Universidade Estadual de Campinas, Faculdade de Ciências Farmacêuticas, Campinas, SP, Brasil; 3 Ministério da Saúde, Secretaria de Vigilância em Saúde e Ambiente, Coordenação-Geral de Editoração Técnico-Científica em Vigilância em Saúde, Brasília, DF, Brasil

A revisão por pares é um dos pilares da qualidade e credibilidade da ciência. Apesar de sua relevância, o processo é imperfeito e é afetado por variabilidade na qualidade e atrasos. O processo de revisão por pares exige gerenciamento constante de vieses e inconsistências. Alguns fatores influenciam diretamente na qualidade das publicações, como a triagem editorial e o número de revisores ^1^. Revisões adicionais após o aceite podem reforçar o rigor científico, mas também frustram as expectativas dos autores. Revisões detalhadas são valorizadas pela percepção de qualidade que proporcionam ^2^, embora o excesso de etapas atrase a publicação ^3^.

Processos editoriais ineficazes geram atrasos injustificáveis, como a demora de semanas para comunicações iniciais por algumas revistas ^4^. A comunicação muitas vezes é prejudicada pela responsabilidade difusa entre editores, o que leva a ruídos na interação com os autores ^5^. A redução de prazos, a melhoria na comunicação e o retorno construtivo aumentam a satisfação dos autores ^6^. A qualificação do processo de revisão por pares busca reduzir essas assimetrias de informação, mitigar a sobrecarga dos revisores e reconhecer o valor de quem contribui efetivamente para o processo editorial.

Para aprimorar o processo de revisão por pares na Epidemiologia e Serviços de Saúde: revista do SUS (RESS) desenvolveu-se um *checklist* com itens hierarquizados para orientar a construção dos pareceres e organizar a avaliação dos manuscritos submetidos à RESS ^7^. O emprego de ferramentas como essa na avaliação por pares potencialmente aumenta a transparência e sistematiza o processo ^8^ ^,^ ^9^.

Para construção do *checklist*, partimos das principais razões de rejeição de artigos científicos submetidos a periódicos ^10^ e revisão da literatura a fim de identificar experiências prévias que fornecessem a estrutura básica ^11^ ^-^ ^18^. A participação dos autores em eventos como o *International Congress of Peer Review and Scientific Publication* (https://peerreviewcongress.org) também forneceram elementos para amadurecer a ferramenta.

O *checklist* foi estruturado em itens críticos, importantes e desejáveis ^7^ que auxiliam o parecerista a avaliar se a pesquisa atende a requisitos essenciais, como a relevância da pergunta para o Sistema Único de Saúde (SUS), a adequação do delineamento para a investigação proposta, a coerência dos métodos - epidemiológicos, estatísticos ou qualitativos - com o objetivo, a consistência e robustez dos resultados com os métodos empregados e a compatibilidade da conclusão em relação à pergunta norteadora e às diretrizes do SUS. A expectativa é que sua adoção sistemática beneficie revisores, editores, autores e leitores, organizando a revisão de forma a reduzir o viés e o tempo de avaliação.

A fim de testar o *checklist* e treinar revisores e editores, organizamos a primeira ‘RESSatona: maratona de revisão por pares da RESS’ em junho de 2024, em Brasília, que ocorreu por meio de projeto de extensão da Universidade de Brasília ^19^. Maratonas de revisão semelhantes foram relatadas para capacitar estudantes de pós-graduação, engajando-os em processos de revisão e aprimorando suas competências em avaliação de artigos ^20^. Outras duas maratonas foram realizadas em 2024, totalizando 96 pesquisadores treinados na prática de revisão por pares ^21^ ^,^ ^22^. Essas maratonas podem ser replicadas em outras instituições interessadas em fortalecer a comunicação científica entre pesquisadores, docentes e discentes de pós-graduação, bem como aproximar a comunidade acadêmica da RESS e sua equipe. Além de contribuir com os periódicos, atuar como parecerista promove a formação de competências críticas e concorre para consolidar a carreira de cientistas ^23^. Os esforços relatados buscam fortalecer o processo editorial da RESS, sistematizando-o para ser cada vez mais justo, eficaz e alinhado aos interesses do SUS.
